# Creating a Natural Vascular Scaffold by Photochemical Treatment of the Extracellular Matrix for Vascular Applications

**DOI:** 10.3390/ijms23020683

**Published:** 2022-01-08

**Authors:** Katalin Kauser, Kevin S. Warner, Blake Anderson, Edgar Dalles Keyes, RB Hayes, Eric Kawamoto, DH Perkins, Robert Scott, Jim Isaacson, Barb Haberer, Ann Spaans, Ronald Utecht, Hank Hauser, Andrew George Roberts, Myles Greenberg

**Affiliations:** 1 Alucent Biomedical Inc., 675 Arapeen Dr, Suite #102, Salt Lake City, UT 84108, USA; KWarner@alucentbiomedical.com (K.S.W.); BAnderson@alucentbiomedical.com (B.A.); RHayes@alucentbiomedical.com (R.H.); EKawamoto@alucentbiomedical.com (E.K.); DPerkins@alucentbiomedical.com (D.P.); RScott@alucentbiomedical.com (R.S.); JIsaacson@alucentbiomedical.com (J.I.); HHauser@alucentbiomedical.com (H.H.); MGreenberg@alucentbiomedical.com (M.G.); 2 Department of Chemistry, University of Utah, 315 South 1400 East, Salt Lake City, UT 84112, USA; u0764411@utah.edu (E.D.K.); Roberts@chem.utah.edu (A.G.R.); 3 Alumend, LLC, 4800 N. Career Avenue, Suite #108, Sioux Falls, SD 57107, USA; Barb.Haberer@alumend.com (B.H.); Ann.Spaans@alumend.com (A.S.); Ron.Utecht@alumend.com (R.U.)

**Keywords:** natural vascular scaffolding, photoactivated linking, extracellular matrix, vascular wall

## Abstract

The development of bioscaffolds for cardiovascular medical applications, such as peripheral artery disease (PAD), remains to be a challenge for tissue engineering. PAD is an increasingly common and serious cardiovascular illness characterized by progressive atherosclerotic stenosis, resulting in decreased blood perfusion to the lower extremities. Percutaneous transluminal angioplasty and stent placement are routinely performed on these patients with suboptimal outcomes. Natural Vascular Scaffolding (NVS) is a novel treatment in the development for PAD, which offers an alternative to stenting by building on the natural structural constituents in the extracellular matrix (ECM) of the blood vessel wall. During NVS treatment, blood vessels are exposed to a photoactivatable small molecule (10-8-10 Dimer) delivered locally to the vessel wall via an angioplasty balloon. When activated with 450 nm wavelength light, this therapy induces the formation of covalent protein–protein crosslinks of the ECM proteins by a photochemical mechanism, creating a natural scaffold. This therapy has the potential to reduce the need for stent placement by maintaining a larger diameter post-angioplasty and minimizing elastic recoil. Experiments were conducted to elucidate the mechanism of action of NVS, including the molecular mechanism of light activation and the impact of NVS on the ECM.

## 1. Introduction

The use and need for improved bioscaffolds in today’s medicine have increased dramatically with modern technology. However, it remains a challenge in cardiovascular tissue engineering to develop biomaterials which can simultaneously substitute for the biological and biomechanical functions of the natural extracellular matrix (ECM) of blood vessels. The ECM is a complex combination of structural proteins including collagen, elastin, fibronectin, laminin and functional proteins such as glycosaminoglycans and growth factors [1]. In the blood vessel wall, ECM serves important structural and functional roles critical for the maintenance of vascular homeostasis and regeneration. It is a key regulator of cellular functions including phenotypic stability, proliferation and apoptosis of vascular cells [1]. Therefore, dysfunctional or damaged ECM is a source of pathological changes [2,3,4,5,6,7] and, not surprisingly, it is difficult to mimic the natural ECM by synthetic bioscaffolds [8].

It is apparent that components of the endogenous ECM can substitute best for the properties of the native tissue [3,4]. Native ECM biomechanics can be modified by changes in covalent bond rearrangement or crosslinking between proteins of matrix components. Different crosslinking approaches, mostly using chemical crosslinking agents, have been evaluated, but all were associated with undesirable side effects such as inflammation or the induction of calcification and suboptimal diffusion pattern [9]. An alternative method is a light irradiation-induced crosslinking process which does not introduce any toxic chemicals and minimizes the damage to structural proteins. Natural vascular scaffolding (NVS) therapy is based on this technology by using a novel, small molecule photosensitizer, 10-8-10 Dimer, to facilitate the photoactivated linking of native collagen and elastin using 450 nm light to create a natural vascular scaffold in the vessel wall.

While the photoactivated 10-8-10 Dimer catalyzed covalent bond formation between oxidizable amino acids fits into the category of molecular crosslinking [10], it is important to distinguish this reaction from the crosslinking of tissues as a result of chemical crosslinkers for tissue fixation [11], the pathological crosslink formation in atherosclerotic arteries as a result of decades-long exposure to oxidants [12] and from other therapies based on similar mechanisms but exposing mostly decellularized tissues for prolonged photoactivated treatment [13]. The actual mechanisms of photochemical protein crosslinking are not easily identified because of the wide variety of possible reactive species and conformations found in proteins. For these reasons, photochemical crosslinking mechanisms have been mostly studied using model systems such as amino acids or small peptide sequences instead of whole proteins [14].

In the present article, the presumed mechanism of action of the NVS technology is discussed. To demonstrate that non-specific crosslinking of proteins did not compromise the cellular viability of the treated vascular segment where the benefit of scaffolding is expected to be most significant, a series of in vitro and in vivo experiments were conducted with ancillary histological and imaging evaluations.

## 2. Results

### 2.1. Pentapeptide Crosslinking Studies to Elucidate the Scaffolding Effect

Photoactivation of 10-8-10 Dimer results in the formation of excited species that can initiate inter and intra formation of covalent bonds in proteins. A series of experiments were conducted previously by Keyes et al. [10], wherein a photochemical mechanism was proposed. The proposed mechanism results in covalent bond formation within and between proteins when exposed to the 10-8-10 Dimer in the presence of light at its absorption maximum. This mechanism is described briefly below.

**Figure 1:**
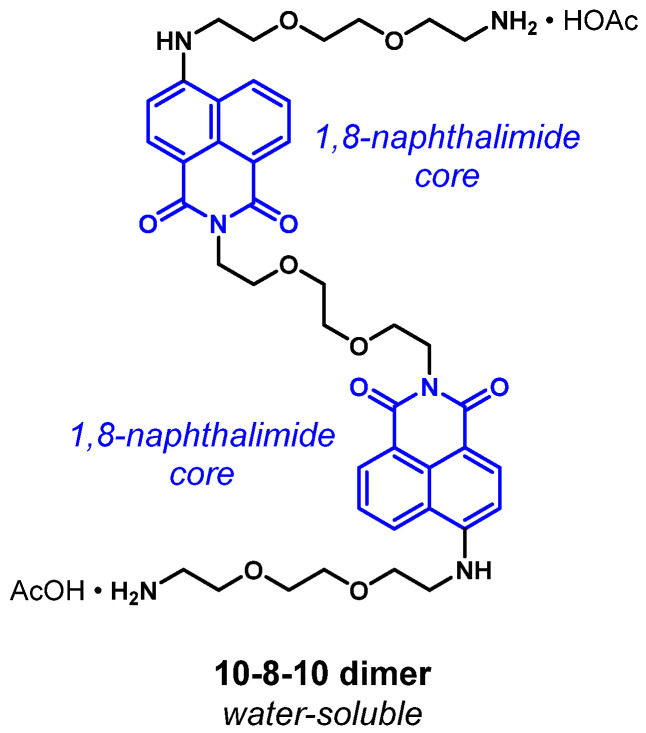
Structure of 10-8-10 Dimer.

**Figure 2:**
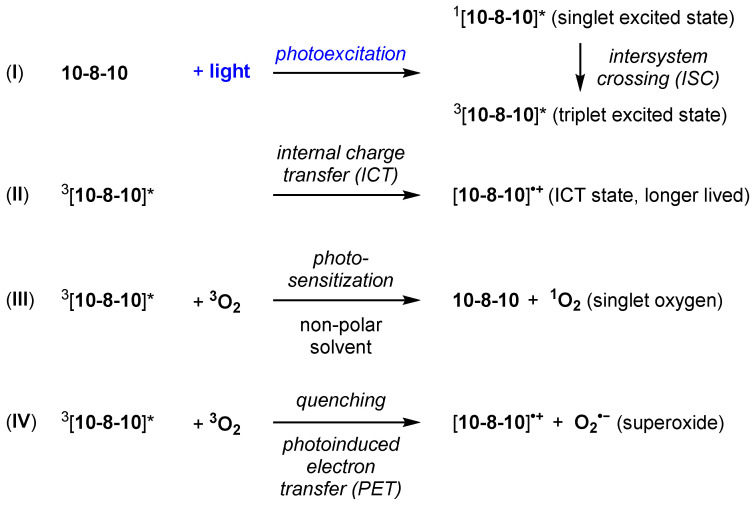
Proposed 10-8-10 Dimer photoexcitation events (I,II) and promoted reactions to generate oxidants (III,IV).

As described by Kelly et al. [15], the 4-amino substituted 1, 8-naphthalimide core of 10-8-10 Dimer (Figure 1) is proposed to undergo photoexcitation upon blue-light illumination (450 nm) to generate a transient singlet excited state, ^1^[10-8-10]*, which converts via intersystem crossing (ISC) to a triplet excited-state intermediate, ^3^[10-8-10]*, (Figure 2, Equation (I)). The triplet excited-state intermediate ^3^[10-8-10]* can proceed to a water-stabilized and longer-lived intramolecular charge transfer (ICT) state, [10-8-10]^•+^ (Figure 2, Equation (II)). This longer-lived ICT state, [10-8-10]^•+^, is not affected by dissolved oxygen. The model reactivity in non-polar solvent, describing ^3^[10-8-10]* as a triplet sensitizer, is consistent with the ability of related triplet sensitizers to convert triplet oxygen into singlet oxygen (Figure 2, Equation (III)). Model reactivity and photophysical studies with 10-8-10 in polar solutions also provide evidence for the ability of the triplet excited-state intermediate, ^3^[10-8-10]*, to reduce methyl viologen via photoinduced electron transfer. Because the one-electron reduction potential of triplet molecular oxygen is more positive than that of methyl viologen (E(O_2_/O_2_^•−^) = −0.16 V vs. NHE), this reductive formation of superoxide is reasonable (Figure 2, Equation (IV)).

**Figure 3:**
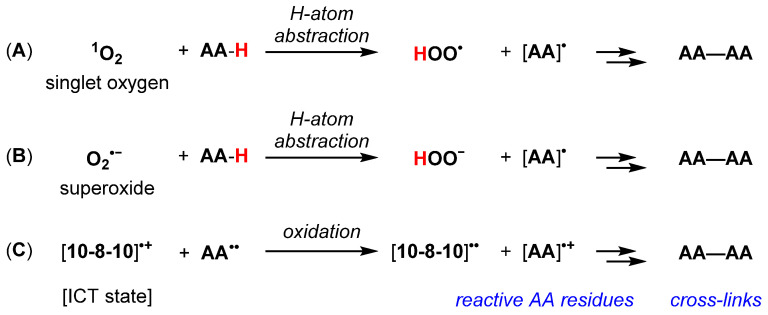
(**A**–**C**) Proposed 10-8-10 Dimer promoted reactions to generate oxidized amino acids [AA]* intermediates and subsequent crosslinks (AA—AA).

**Figure 4:**
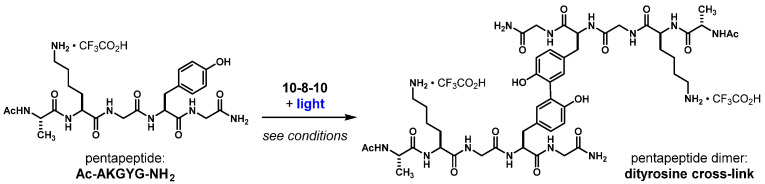
Pentapeptide dimerization reaction.

Accordingly, the illumination of the 10-8-10 dimer could affect photosensitized oxidation and subsequent crosslinking reactions at oxidizable amino acid residues (e.g., tyrosine, tryptophan, histidine, methionine, cysteine) (Figure 3A). Mechanisms involving superoxide (O_2_^•−^, Figure 3B) to affect H-atom abstraction have been described [15,16,17]. However, in polar, aqueous media, ICT state-promoted [10-8-10]^•+^ oxidation of amino acid residues cannot be ruled out (Figure 3C). These reaction mechanisms are supported by the reaction scheme of a pentapeptide (Ac-AKGYG-NH_2_)-containing tyrosine exposed to 10-8-10 Dimer and light at the wavelength absorption maximum which resulted in covalently bond dityrosine as a crosslinked product (Figure 4).

To support the above-described experiments using exposures estimated for future clinical applications, studies were conducted to identify if tyrosine residues would undergo oxidation to form tyrosyl radical intermediates and dityrosine formation when exposed to various 10-8-10 dimer concentrations and irradiation (light dose). A range of 10-8-10 dimer concentrations, relative to the model pentapeptide (expressed as mole ratio of 10-8-10 Dimer to pentapeptide), were selected to bracket the same mole ratio range of 10-8-10 Dimer to elastin and collagen estimated in arteries [18].

**Figure 5:**
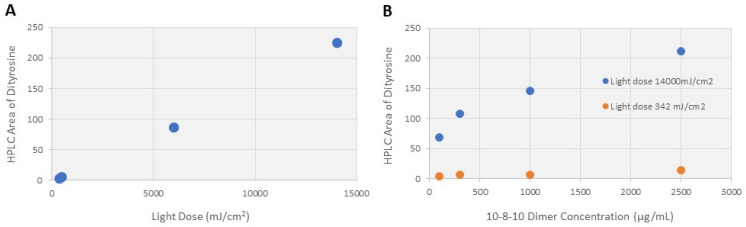
(**A**) Formation of dityrosine with varying light dose. 10-8-10 Dimer and model peptide concentrations were in excess and kept constant. (**B**) Formation of dityrosine as function of 10-8-10 Dimer concentration at 2 light doses.

Figure 5A summarizes the amount of dityrosine (represented by HPLC area) formed as a function of light dose, and Figure 5B summarizes the amount of dityrosine formed as a function of 10-8-10 Dimer concentration at the minimum (342 mJ/cm^2^) and maximum (14,000 mJ/cm^2^) light doses studied. The proposed mechanism of crosslinking formation and results from the pentapeptide dimerization study provide an explanation for the effect measurements reported in Section 2.2, Section 2.3 and Section 2.4 below.

### 2.2. Scaffolding Effect Quantitated by Luminal Gain Retention Using Porcine Carotid Arteries In Vitro

Fresh porcine carotid artery segments were treated with different concentrations of 10-8-10 Dimer solution and a set dose of light for 5 min under overstretch (created using an angioplasty balloon inflated to a diameter to induce the desired overstretch). Retention of luminal gain (diameter retention) was calculated and reported as % luminal gain in comparison to arteries treated with 10-8-10 Dimer and light at the time of overstretch versus arteries treated with angioplasty balloon overstretch only (Table 1). Based on these experiments, a concentration of 10-8-10 Dimer above 65 ng/mg and light dose of 11–13 J/cm^2^ was needed to achieve the NVS effect.

### 2.3. Effect of Vascular Scaffolding on Arteries Treated In Vitro

#### 2.3.1. Studies Using Multiphoton Microscopy Imaging

**Figure 6:**
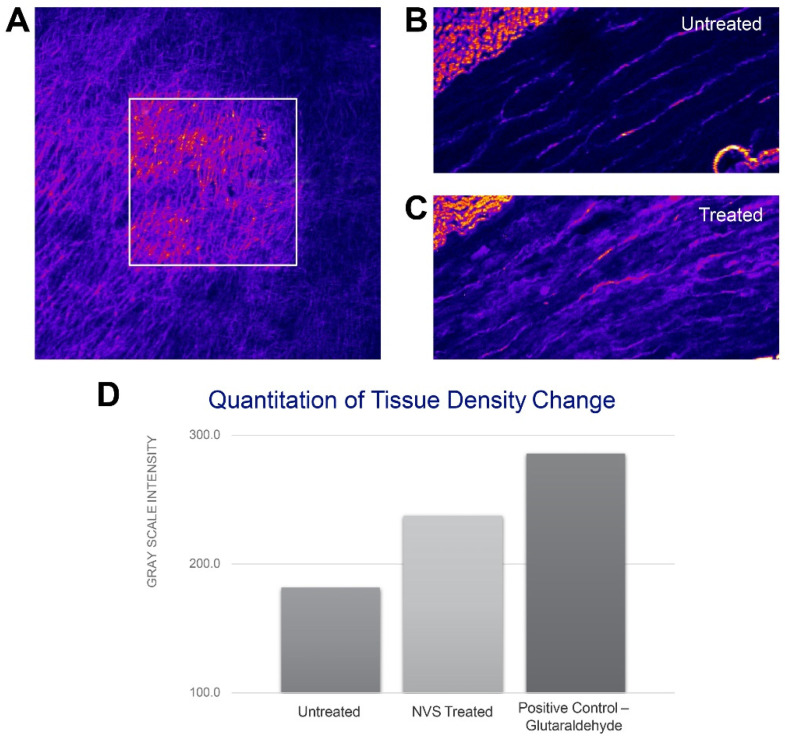
Visualization of NVS treatment effect on arterial wall segment by multiphoton microscopy imaging: (**A**) Second harmonic generation showing collagen change as a result of photoactivation (square area). Representative histological cross-sections of untreated (**B**) and NVS-treated tissue (**C**). (**D**) Quantitation of tissue density change as determined by gray scale intensity between untreated (**left** bar), NVS- (**middle** bar) and glutaraldehyde-treated (**right** bar) tissue.

Differences in collagen fibrillarity can be visualized with second harmonic generation (SHG) using multiphoton microscopy imaging [19]. This method was tested to determine its capability of visualizing differences in NVS-treated tissue in comparison to naïve tissue. Figure 6A shows a porcine carotid artery segment previously soaked in the 10-8-10 Dimer solution and exposed to the 450 nm light (squared area). Compared with non-treated tissue, arteries treated with 10-8-10 Dimer solution and light (NVS treatment) resulted in a change in the intensity of the collagen signal which indicates the photochemical crosslinking effect. Representative histological cross-sections of untreated and NVS-treated arteries are shown in Figure 6B,C, respectively. Compared to untreated arteries, NVS treatment resulted in increased ECM fibrillarity. Figure 6D shows the gray scale intensity of untreated, NVS- and glutaraldehyde-treated arteries (positive control). Overall, the results indicate that the photoactivated crosslinking effect can be distinguished from tissue density caused by chemical crosslinking in the tunica media and clearly differentiated from the untreated artery.

#### 2.3.2. Biomechanical Measurements

**Figure 7:**
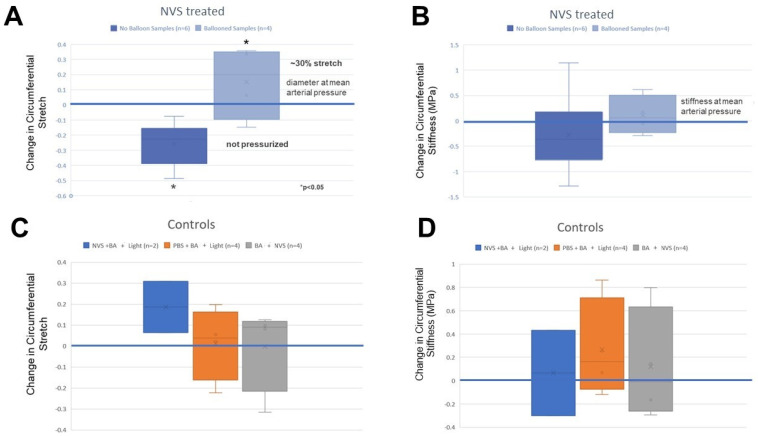
NVS treatment effect on vessel diameter (**A**) and vessel stiffness (**B**) with their respective control studies performed with the balloon-treated arteries (Panels (**C**,**D**)). The full treatment was compared to balloon and light or balloon and 10-8-10 Dimer treatment alone.

Biaxial mechanical tests were conducted using fresh NVS-treated porcine carotid arteries while stretched open with an intraluminal balloon or left without intraluminal pressurization. The differences in both stretch and stiffness were calculated during pressure cycles between 6.7–22 kPa. As shown in Figure 7A, NVS treatment without balloon dilation (n = 6) resulted in a decrease in the operational diameter of −0.112 ± 0.267, while treated segments with balloon dilation (n = 4) had a significant increase in diameter (+0.140 ± 0.206; Student *t*-test, *p* ˂ 0.05). However, NVS treatment with or without balloon dilation was similar between groups (Student *t*-test, *p* = NS) indicating that, although NVS treatment produced lasting changes in luminal gain, it did not change stiffness (Figure 7B).

### 2.4. Effect of Vascular Scaffolding on Porcine Arteries Treated In Vivo

#### 2.4.1. Acute Studies in Comparison to Plain Old Balloon Angioplasty

**Figure 8:**
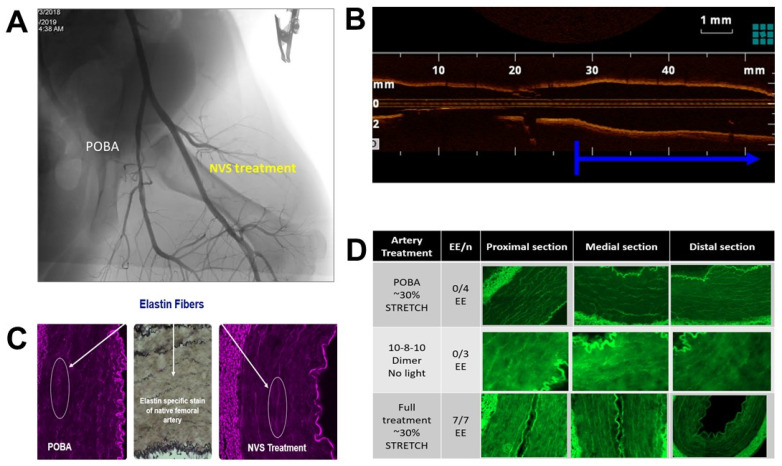
NVS treatment effect in healthy swine: (**A**) Angiographic image of the scaffolded or NVS- and plain old balloon angioplasty (POBA)-treated arteries. (**B**) Longitudinal intravascular optical coherence tomography image of the NVS-treated artery (blue arrow) in comparison to the adjacent non-inflated and treated part of the same artery. (**C**) Elastin fibers after POBA and NVS treatment (white arrows pointig to areas in white oblongs). (**D**) Histological cross-sections from POBA and only 10-8-10 Dimer-treated vessels in comparison to the section from NVS-treated vessels.

Femoral and iliac arteries of healthy swine were treated with NVS under 30% overstretch to examine the effect the scaffolding treatment on retaining the stretched lumen size. Figure 8 shows the angiographic (A) and optical coherence tomography (OCT) images (B) of the treated vessels in comparison to plain old balloon angioplasty (POBA) or the adjacent untreated vascular segment. As predicted, NVS treatment during overstretch balloon dilation led to a shape change as confirmed by angiography, OCT and histology. Immediately following treatment, the arteries were collected into liquid nitrogen for the histological examination of the ECM. As a result of the scaffolding treatment applied under stretch, morphological changes of the elastin and collagen fibers were observed in the tunica media and the internal elastic lamina of the tunica interna of the treated arteries (Figure 8C). The elastin fibers, which naturally appear coiled in a histological cross-section of an artery, remained elongated. This elongated elastin phenomenon was present in all fully treated vessels examined, but not in control and only 10-8-10 Dimer-treated vessels without the light activation (Figure 8D). Elongated elastin corresponded with the scaffolding-caused shape change pictured by angiography or OCT imaging and represented the inability of the elastin fibers to return to their recoiled state due to the scaffolding treatment caused by newly formed covalent bounds.

#### 2.4.2. Chronic Studies in Comparison to Plain Old Balloon Angioplasty and Stent Treatment in Diseased Arteries

**Figure 9:**
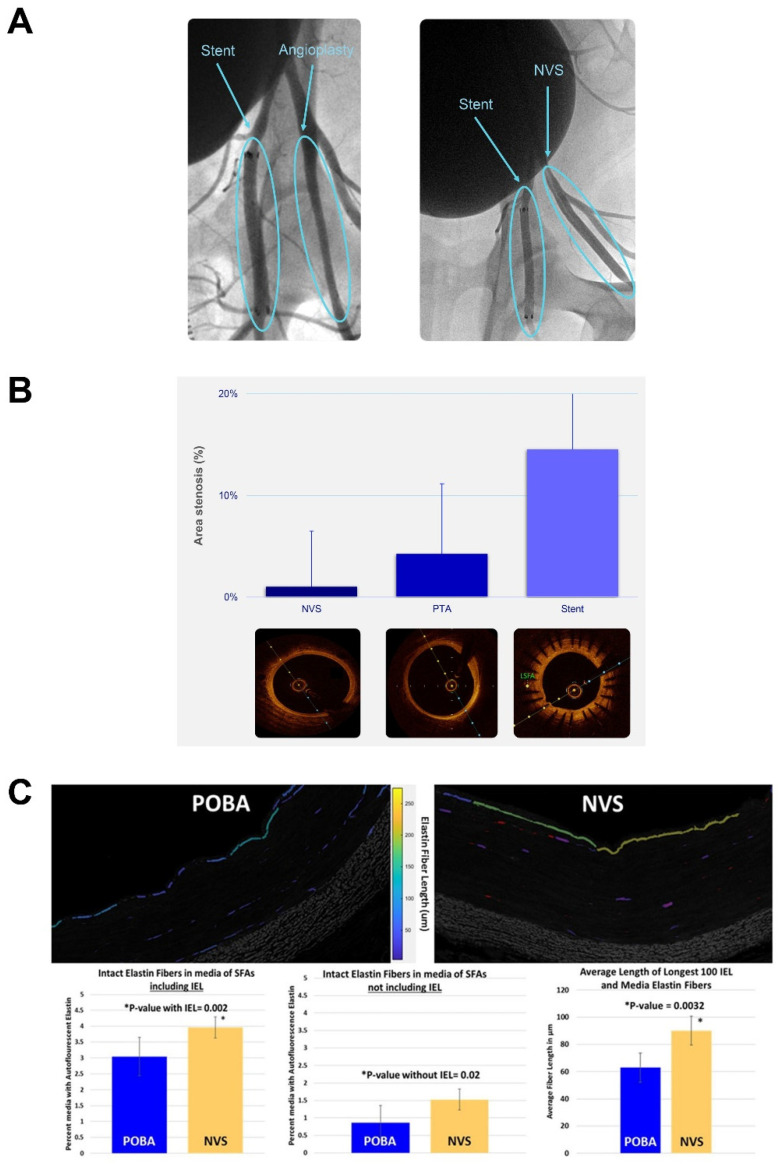
NVS treatment effect in hypercholesterolemic swine: (**A**) Angiographic images of stent- and POBA-treated vessels (**left** panel) and stent- and NVS-treated vessels (**right** panel). (**B**) Optical coherence tomography images of the NVS-scaffolded vessels in comparison with POBA and stenting. (**C**) Multiphoton images of arterial cross-sections after NVS and POBA treatment with quantitated elastin fiber amount and length in the vessel wall using MIPAR analysis.

In order to understand the performance of this photochemically initiated scaffold in a disease-like tissue environment, NVS treatment was performed in a model of atherosclerosis using genetically modified, hypercholesterolemic miniature swine (ExeGen LDLR MiniSwine) [20]. NVS treatment was compared to both stenting and POBA, and the vessels were analyzed after necropsy 28 days following treatment. Angiographic images taken acutely following treatment demonstrated that the NVS-treated segments retained their expanded shape similar to stents as observed in normal swine (Figure 9A). OCT images revealed that the NVS-treated vessels had not lost lumen size, whereas the stented arteries became narrower. A similar trend was also seen in the only angioplastied vessels (Figure 9B). Histological examination confirmed intimal hyperplasia was associated with stent placement, which was not observed after NVS treatment (data not shown). In addition, the photochemical scaffolding resulted in significantly preserved elastin content in the vessels compared to the untreated POBA alone control. Besides the amount of preserved elastin, the length of the detected fibers was also significantly longer (Figure 9C).

## 3. Discussion

Peripheral arterial disease (PAD) of the lower extremities, the narrowing of blood vessels, is primarily due to the presence of atherosclerotic plaque. According to recent estimates, approximately 8.5 million Americans > 40 years of age [21] and more than 236 million individuals worldwide [22] have various forms of the disease. In addition to the progressive decline in ambulatory function and quality-of-life, individuals with PAD are also at increased risk of cardiovascular mortality and morbidity of the same magnitude as seen in patients with cardiovascular disease [23].

Revascularization of the ischemic limb is the cornerstone of guideline-directed medical therapy [24,25]. Although minimally invasive endovascular procedures with self-expanding stents have proven effective [26], the presence of a permanent scaffold within the vessel wall promotes inflammation, disturbs flow dynamics, abolishes vascular reactivity and often leads to the development of in-stent restenosis [27]. Bioresorbable scaffolds have been explored as a solution to provide early structural support with the recovery of vasomotor function following absorption [28,29,30]. Although effective in the short-term, long-term studies have been hampered by increased rates of stent thrombosis compared with conventional stents [31,32]. New treatment modalities are required to address this medical need. It is noteworthy the lower extremity arteries have complex biomechanics with extensive mechanical deformation, twisting, bending and compression of the major vessels with each leg movement [33,34,35,36]. Because of this and the less compliant nature of conventional metal stents, kinking and strut fractures often occur and are thought to play major roles in vessel wall injury and flow disturbance, key factors in the development and progression of restenosis [37,38,39].

The present studies investigated the potential benefit of NVS treatment at the time of balloon angioplasty to maintain the lumen diameter gain of the dilated vessel. As there is no rigid scaffold, the arterial wall would theoretically retain its natural functionality and flexibility, thereby avoiding the complications of conventional stents. Moreover, by preventing elastic recoil, NVS has the potential to limit the development of restenosis, the main limitation of balloon angioplasty. Decreasing the rate of restenosis should translate to higher long-term primary patency rates, and therefore, better patient mobility, reduced pain and improved quality of life. Systematic experiments were conducted to understand the photochemical mechanism of action of 10-8-10 Dimer when exposed to light at its absorption maximum [10,15]. These experiments support that the 4-amino substituted 1,8-naphthalimide core of the 10-8-10 Dimer undergoes photoexcitation upon illumination by a 450 nm light to generate a transient, reversible, excited state of the molecule (Figure 2 and Figure 3). Studies support that the 10-8-10 Dimer in this transient, reversible, excited state facilitates a crosslinking reaction at oxidizable amino acid residues (e.g., tyrosine, tryptophan, histidine, methionine, cysteine) of the three-dimensional structure of the ECM in the vascular wall. It is this crosslinking of amino acid residues in the ECM proteins of an artery that creates the NVS effect. Exploration of the 10-8-10 Dimer and light dose range (Figure 5) capable of eliciting this crosslinking effect measured by dityrosine formation in a simple experimental system is a continuation of the study by Keyes and colleagues [10] to understand the reaction kinetics of the formation of amino acid crosslinking.

Results of biomechanical studies in porcine carotid arteries demonstrate that light activation of 10-8-10 Dimer at the time of balloon angioplasty results in the maintenance of the dilated vessel diameter while having no detrimental effect on the compliance of the vessel to pressure-induced expansion. Retaining the increased luminal diameter suggests that the NVS treatment likely inhibited acute elastic recoil. These observations are in agreement with previous studies in porcine [40] and human popliteal arteries [41]. The latter study also demonstrated that NVS did not alter the contractile responsiveness of the treated arteries [41].

To determine if the NVS effects observed in vitro would also be observed in vivo, a series of experiments were conducted using a healthy swine model of balloon angioplasty. As with the in vitro studies, treatment with NVS of either femoral or iliac arteries during balloon dilation resulted in a greater luminal diameter than balloon angioplasty alone. The histological evaluation of NVS-treated segments showed morphological changes of the elastin and collagen fibers in both the tunica media and internal elastic lamina of the tunica interna. The elastin fibers, which appear naturally coiled, are elongated following treatment. This observation suggests NVS treatment directly interfered with elastin fibers and prevented their natural ability to return to their recoiled state, likely due to newly formed covalent bounds. As elastin fibers are the main mediators of elastic recoil [42,43], interventions aimed at minimizing recoil following balloon angioplasty are clinically relevant. Using an ex vivo model of human tibial artery angioplasty, Burke and colleagues [44] demonstrated that treatment with an elastase inhibitor, which reduced the elastin content by 60%, resulted in an increased vessel artery diameter. However, loss of elastin could lead to pathological consequences. Hypertension, diabetes, obesity and atherosclerosis are generally associated with elastic fiber degradation or detrimental biochemical modification. This degradation initiates destructive arterial remodeling that is difficult to stop or to reverse, such as aneurysm development [45]. Elastin has an estimated 70-year half-life. Therefore, a treatment which retards the process of elastin degradation, such as NVS therapy, could offer patients durable clinical effects. Although this study suggests an NVS-mediated inhibition of elastin recoil, the mechanisms leading to luminal diameter retention are likely complex and involve NVS-mediated crosslinking between different constituents of the ECM. Natural vascular scaffolding appears to both protect elastin and retain its stretch-elongated shape. These effects allow for the opening of the vessel diameter and restoration of blood flow to downstream tissues.

Using a hypercholesterolemic swine model, the beneficial effects of NVS treatment observed in vitro were investigated in vivo. Hypercholesterolemic swine exhibit a robust inflammatory and neointimal response to percutaneous stent placement both in the coronary and peripheral arteries [46], resembling advanced atherosclerotic disease in humans. Compared to NVS treatment and conventional balloon angioplasty, stenting of the profunda femoris and superficial femoral arteries was associated with reduced vessel patency and increased restenosis. NVS treatment and conventional balloon angioplasty resulted in better outcomes. Compared to balloon angioplasty, arteries following NVS treatment had a significantly greater elastin content, suggesting the newly formed covalent bonds exerted protection against extracellular matrix protein degradation as a result of the photochemical scaffolding. Overall, NVS treatment was not associated with the occurrence of adverse changes or appreciable inflammatory response, consistent with the results of previous studies [41,47].

In conclusion, our results suggest the photochemically induced natural scaffolding is capable of durably retaining vascular lumen size with natural flexibility and vascular cellular function. We postulate that this technique will allow for subsequent therapeutic vascular remodeling, without interfering with cell proliferation or creating adverse, rigid vessel biomechanics. Animal studies have shown acute and long-term safety and patency without the pro-inflammatory and mechanical risks of placing a rigid foreign implant into the blood vessel. The safety and efficacy of NVS therapy while facilitating the retention of acute luminal gain during percutaneous transluminal angioplasty will be evaluated under study [NCT04188262] in patients with de novo superficial femoral and proximal popliteal artery lesions. The study completion is expected in August 2022.

## 4. Materials and Methods

### 4.1. Pentapeptide Crosslinking Studies

10-8-10 Dimer was synthesized by Alucent Biomedical, and pentapeptide Ac-AKGYG-NH_2_ was synthesized in Dr. Andrew Roberts’ lab at the University of Utah. Specified concentrations of 10-8-10 Dimer solution and pentapeptide were mixed with PBS buffer and exposed to 450 nm light for a pre-specified duration to achieve light dose exposure. After light exposure, the light source was turned off, and an aliquot was taken and analyzed for presence of dityrosine using an HPLC method.

Table 2 summarizes the amounts of the 10-8-10 Dimer and pentapeptide along with light dose studied. The reaction among 10-8-10 Dimer, light and peptide is summarized in Figure 4.

### 4.2. Luminal Gain Retention

Porcine carotid arteries (n = 50), obtained from a slaughterhouse (Smithfield Foods, Sioux Falls, SD, USA), were cleaned of adherent tissues and placed in fresh phosphate-buffered saline (PBS). A small segment of the test artery was cut and sized by measuring the diameter to determine the balloon the diameter required to attain desired overstretch, and the remaining artery segment was soaked from the luminal surface in a solution of 10-8-10 Dimer for 5 min. The artery was then removed from the 10-8-10 Dimer solution, and an angioplasty balloon with light fiber inserted into the guidewire lumen was introduced into the artery and the balloon to a pre-determined diameter to attain overstretch. Once the diameter was attained, the light source was turned on, illuminating the treatment area with 450 nm light and was left on for 60 s to deliver the desired light dose. At the treatment completion, the balloon was deflated, the catheter removed and the artery placed in PBS solution for 15 min prior to measurement of the lumen diameter. Retention of luminal gain (diameter retention) was calculated and reported as % luminal gain between arteries treated with 10-8-10 Dimer and light at time of overstretch versus arteries treated with angioplasty balloon overstretch only.

### 4.3. Multiphoton Microscopy Imaging

To confirm the usefulness of SHG imaging to demonstrate density changes in the ECM, fresh carotid arteries from 6–9-month-old swine were obtained from Animal Technologies (Tyler, TX, USA), cleaned of adherent tissues and gently flushed to remove any blood clots before being cut open longitudinally and attached to a glass slide. A total of 3 mL of a 2 mg/mL 10-8-10 solution was then dripped on the open luminal surface of the artery and allowed to penetrate for 5 min. With the microscope (Ultima In Vivo 2 Photon GaSP, Bruker Prairie) laser set to 800 nm, an area of good collagen fibers was identified. The laser was then tuned to 887 nm, and an optical zoom of 4 × was used to activate a small square-shaped area within the field of view of the 0x zoom, by a 447 nm secondary harmonic photon capable of activating the 10-8-10 Dimer. After activation of 10-8-10 Dimer the laser was tuned back 800 nm with an optical zoom of 0x, and the area was reimaged.

In vitro benchtop and in vivo SHG imaging studies were performed using carotid arteries of swine 6–9 months old obtained from Animal Technologies (Tyler, TX, USA), cleaned of adherent tissues and gently flush to remove any blood clots. Arteries were then randomly assigned to a treatment group. Glutaraldehyde was used as a positive control, and untreated sections of porcine carotids as well as in vivo POBA arteries were used as a negative control. Following treatment, each segment was frozen, cut into 10 µm sections and placed on charged slides. After overnight drying, the slides were placed in a Petri dish and covered with PBS solution 1–2 cm above the slide. Imaging settings for Ultima In Vivo 2 Photon GaSP microscope were as follows: Nikon 16x NA 0.8 objective; laser power 172; laser wavelength of 800 nm; dwell time of 20.8 ns. Representative sections of the ECM were collected at a resolution of 512 × 512 at 12-bit depth.

### 4.4. Biomechanical Measurements

Fresh carotid arteries from 6–9-month-old swine (Animal Technologies Inc., Tyler, TX, USA) were pre-treated with NVS (4 mg/mL in PBS) for 5 min to allow diffusion into the vessel wall. A customized optical catheter with a balloon at its tip was then guided into the lumen to shine a 457 nm laser for 1 min onto the treatment region to activate the NVS and induce crosslinking [48]. Following photoactivation, the vessels were cut into two tubular specimens and randomly assigned to one of 2 groups: stretch group (30% balloon stretch) (n = 4) or no-stretch group (n = 6). The biaxial mechanical tests were conducted using a custom-built pressure myograph device. All samples were preconditioned through 5 pressure cycles between 6.7 and 22 kPa at incrementally greater levels of constant axial stretch up to the in vivo length and 5% beyond that length. The samples were then biaxially loaded in configurations most similar to the in vivo configuration (1.00× axial in vivo stretch and 15.8 kPa luminal pressure).

### 4.5. Acute Studies in Healthy Swine

Yorkshire swine of either sex weighing 30–40 kg (n = 35 in 4–5 separate experimental procedures) were pre-treated with 300 mg of clopidogrel and 250 mg of acetylsalicylic acid prior to percutaneous transluminal angioplasty (PTA). Animals underwent a single acute procedure in which 3–4 vessels per animal (right and left superficial femoral artery and internal iliac artery) were subjected to one of two treatments, NVS therapy or PTA balloon only. Angiography and OCT imaging were performed before device treatment, during and post-PTA inflation and at euthanasia for all animals. Activated clotting times were monitored during the interventional procedures. The treated arteries were harvested freshly, collected in liquid nitrogen and processed for histopathologic evaluation.

### 4.6. Chronic Studies in Hypercholesterolemic Swine

Nine familial hypercholesterolemic swine (ExeGen LDLR Yucatan miniature swine; Exemplar Genetics, Sioux Center, IA, USA) of either sex weighing between 64–97 kg underwent oversized (30%) balloon injury in the bilateral profunda femoris and superficial femoral arteries 13 days (Day 0) before treatment to induce lesions consisting of neointimal proliferation. On day 14, the animals were randomly assigned to one of 3 groups: (1) NVS treatment (4 mg/mL in PBS; n = 3 animals, n = 12 sites), (2) balloon angioplasty (Abbott Armada 35 PTA Balloon Catheter; n = 3 animals, n = 12 sites) or (3) stenting (C.R. Bard E-Luminexx Biliary Stent; n = 3 animals, n = 12 sites). The NVS solution (2 mL) was introduced through the Occlusion Perfusion Catheter balloon (Advanced Catheter Therapies Inc., Chattanooga, TN, USA). Immediately following treatment, angiography and OCT imaging were performed. Four weeks following treatment (Day 42), the animals underwent repeat imaging, were euthanized and the vessels with the treated segments were explanted for histopathology evaluation.

### 4.7. Histology

Routine processing of formalin-fixed, paraffin-embedded histology was completed at Alizee Pathology Inc. (Thurmont, MD, USA). A minimum of 4 pictures, representing 4 quadrants, were captured using “Jenoptik” CCD camera and software. Immunohistochemistry (IHC)-stained formalin-fixed paraffin-embedded (FFPE) blocks were sectioned at 4 µm and stained with mouse anti-elastin using 3,3′-Diaminobenzidine Chromogen. Internal controls were used to determine proper staining quality. Elastin IHC stained slides were imaged at Alucent Biomedical using Olympus BX41 Fluorescent Microscope with a 40× objective. SHG imaging was performed on 10 µm thick FFPE sections using Leica SP8 Dive with spectral tunable detection. SHG images were generated and stitched together using “Leica SP8 Dive with spectral tunable detection” microscope 25×/0.95NA water objective, collagen imaged at 1300 nm, elastin imaged at 1045 nm, power 15,400 watt. “MIPAR Analysis Software” was used for the quantification of elastin via IHC-stained slides. Images of the arteries were analyzed as follows: Color deconvolution, which was used to separate a color image into channels (colors) that are not the basic red, blue and green channels. Color deconvolution was used to separate positively stained elastin fibers (brown), background (light blue) and nuclei (dark blue). Media of the vessel in the region of interest was selected for boundaries. Fibers with positive staining were overlaid onto the region of interest and the percentages calculated. Each artery that was evaluated for its elastin content had their values recorded on an excel sheet and the final calculated data plotted.

### 4.8. Vascular Imaging

Vascular imaging was performed through fluoroscopy and/or OCT. Quantitative vascular analysis was performed fluoroscopically by first establishing a baseline diameter using the guide sheath followed by measurements of the artery diameter before and after NVS treatment. The diameter measurements were taken at three locations along the treatment length (proximal, medial and distal sections) for all pre- and post-NVS treatment data points. This was performed using the fluoroscopy software of the labs (GE Centricity Cardiology CA1000 Cardiac Review 2.0 or Siemens ACOM.PC). OCT imaging was performed with the Dragonfly Duo imaging catheter connected to the Ilumien console (St. Jude Medical, Memphis, TN, USA). The catheter was advanced distal to the treatment site, the catheter pulled back and images recorded via the console. Diameter measurements were taken at the proximal, medial and distal sections of the treatment area.

## Figures and Tables

**Table 1:** Luminal gain measurements using in vitro porcine carotid artery.

10-8-10 Dimer Solution Concentration (mM)	Time of 10-8-10 Dimer Solution Exposure	10-8-10 Loading in Artery (ng/mg) *	Light Dose (J/cm^2^) **	Luminal Gain (Pass Criteria > 16%)
0.15	5 min	NT	11 J/cm^2^–13 J/cm^2^ (188 mW/cm^2^ * 60 s = 11,280 mJ/cm^2^ or 11 J/cm^2^)	8 ± 1
0.3		20		10 ± 1
0.6		65		14 ± 4
1.2		72		18 ± 7
2.5		155		22 ± 5

* 10-8-10 Dimer concentration measured after measurements completed. Measurements represent the mean of at least 3 independent determinations. ** light dose used in these studies corresponds with clinically used light doses.

**Table 2:** Reaction conditions used to study pentapeptide dimerization at tyrosine.

10-8-10 Dimer Solution Concentration (mg/mL)	Molecular Weight 10-8-10 Dimer (Dalton)	Concentration of 10-8-10 Dimer (mmol/mL)	Concentration of Peptide (mmol/mL)	Mole Ratio (10-8-10/Peptide) *	Light Dose (mJ/cm^2^)
0.1	800	0.00013	0.0013	1:10	342
0.3	800	0.00038	0.0013	1:3	342
2.5	800	0.0031	0.0013	1:0.4	14,000

* Ratio is an estimate of a 10-8-10 Dimer to amino acid ratio that could be present in vivo.

## Data Availability

The data presented in this study are available on request from the corresponding author (Katalin Kauser).
